# Anti-MJ/NXP-2 autoantibody specificity in a cohort of adult Italian patients with polymyositis/dermatomyositis

**DOI:** 10.1186/ar3822

**Published:** 2012-04-30

**Authors:** Angela Ceribelli, Micaela Fredi, Mara Taraborelli, Ilaria Cavazzana, Franco Franceschini, Marzia Quinzanini, Angela Tincani, Steven J Ross, Jason YF Chan, Brad A Pauley, Edward KL Chan, Minoru Satoh

**Affiliations:** 1 Department of Oral Biology, University of Florida, 1395 Center Drive, Gainesville, FL 32610-0424, USA; 2 Rheumatology Unit, A.O. Spedali Civili, Piazzale Spedali Civili 1, 25123, Brescia, Italy; 3 Division of Rheumatology and Clinical Immunology, Department of Medicine; Department of Pathology, Immunology, and Laboratory Medicine, University of Florida, 1600 SW Archer Road, Gainesville, FL 32610-0221, USA

## Abstract

**Introduction:**

Autoantibodies in patients with polymyositis/dermatomyositis (PM/DM) are associated with unique subsets, clinical course and outcome. Anti-MJ antibodies, which recognize the nuclear protein NXP-2/MORC3, are reported in ~25% of juvenile DM. Prevalence and clinical significance of anti-MJ antibodies in adult Italian PM/DM patients were studied.

**Methods:**

Sera from 58 consecutive adult Italian PM/DM patients were analyzed by immunoprecipitation of ^35^S-labeled K562 cells extract, ELISA (anti-MJ, Jo-1), Western blot and indirect immunofluorescence. Clinical associations were analyzed using information from medical charts.

**Results:**

Anti-MJ antibodies were the most prevalent specificity (17%) found mainly in DM (30%, 8 cases) vs 8% of PM (2 cases, *P *= 0.02). Comparing 10 anti-MJ (+) vs 48 anti-MJ (-) cases, DM was more common (*P *= 0.03), and age at onset was younger in anti-MJ (+) (*P *= 0.0006). In anti-MJ (+), heliotrope rash (*P *= 0.01) and calcinosis (*P *= 0.09) were more frequent. None of them had heart or lung involvement, or malignancy. Myopathy in anti-MJ (+) patients responded well to therapy and none of them had elevated CPK at last visit (0% vs 25% in anti-MJ (-)). Only 60% of anti-MJ (+) showed immunofluorescent nuclear dots staining, despite PML localization of NXP-2/MORC3.

**Conclusions:**

Anti-MJ antibodies are the most frequent specificity in our cohort of adult Italian PM/DM. Anti-MJ (+) were associated with young onset DM, calcinosis, no internal organ involvement and good response of myopathy to therapy. Anti-MJ reported in juvenile DM is also found in adult PM/DM, and could be a new useful biomarker.

## Introduction

Myositis-specific autoantibodies (MSAs) are helpful in the diagnosis of polymyositis/dermatomyositis (PM/DM), in identifying distinct clinical subsets, and disease monitoring [1-3]. MSAs, including antibodies to aminoacyl-tRNA synthetases (Jo-1 is the most frequent), anti-p155/140, -CADM-140, -Mi-2, and -SRP are generally found in ~50% of adults with PM/DM [3]. Since many patients do not have known MSAs, it is important to characterize additional autoantibodies in PM/DM, and to clarify their clinical significance. A new autoantibody, called anti-MJ, has been identified in juvenile DM (JDM) patients [4,5], and it was associated with severe muscle weakness, polyarthritis, joint contractures, and intestinal vasculitis [4]. In a cohort of Argentine pediatric myositis patients, anti-MJ antibodies were the most prevalent specificity (25% of cases), associated with muscle contracture, atrophy and significant compromise of the functional status [6]. The target of anti-MJ antibodies was identified as a nuclear protein called NXP-2 [7], which plays important roles in diverse nuclear functions, including RNA metabolism and maintenance of nuclear architecture [7]. NXP-2 (also known as MORC3) [8,9] localizes in the PML (promyelocytic leukemia) nuclear bodies, where it recruits and activates p53 to induce cellular senescence [8,10]. The presence of anti-MJ antibodies in adult patients with myositis has been described in an abstract analyzing a British cohort [11] and in a recent paper showing a possible link between anti-MJ antibodies and higher risk of malignancy in a Japanese cohort of adult PM/DM patients [12]. It is also still unclear whether anti-MJ is found in adult PM/DM has same clinical significance as JDM. The aim of our study is to analyze the prevalence and clinical significance of anti-MJ antibodies in a cohort of adult Italian PM/DM patients.

## Materials and methods

### Patients

Fifty-eight consecutive adult Italian patients with PM/DM, who visited the Rheumatology Unit of Spedali Civili (Brescia, Italy) from 2009 to 2011, were studied. The diagnosis of PM/DM was based on Bohan and Peter criteria [13]. Clinical information was obtained from medical records. The study was approved by the Institutional Review Board of the hospital. This study meets, and is in compliance with, all ethical standards of medicine, and informed consent was obtained from all patients in accordance with the Declaration of Helsinki.

### Immunoprecipitation (IP)

Autoantibodies in sera were screened by IP using ^35^S-methionine-labeled K562 cell extract [14]. Specificities of autoantibodies were determined using reference sera.

### ELISA

Anti-MJ antibodies were also tested by antigen-capture ELISA [15]. Briefly, a 96-well plate Immobilizer Amino (Nalge Nunc International, Rochester, NY, USA) was incubated with 50 μl/well of 2 μg/ml mouse monoclonal antibody (mAb) to MORC3 (MBL International, Woburn, MA, USA). Wells were then incubated with K562 cell extract, followed by 1:500 diluted sera, and probed with alkaline phosphatase conjugated mouse mAb anti-human Immunoglobulin G (1:2000 dilution) (Sigma, St. Louis, MO, USA). Anti-Ro52, -La [16,17], and -Jo-1 (Abazyme, Needham, MA, USA) antibodies were tested by ELISA using recombinant protein at 1:500 serum dilution. Optical density (OD) of the samples was converted into units using a standard curve created by a prototype positive serum [15].

### Immunoprecipitation - Western Blot (IP-Western)

Candidates for anti-MJ were selected based on immunoprecipitation of a 140 kDa protein that comigrates with MORC3 protein recognized by mAb. Identity of the 140 kDa proteins corresponding to MJ/MORC3/NXP-2 was verified by IP-Western [14]. Cell extract from 10^7 ^K562 cells was immunoprecipitated by candidate sera. Proteins were fractionated by 8% sodium dodecyl sulfate-polyacrylamide gel electrophoresis (SDS-PAGE) and transferred to a nitrocellulose filter. The filter was probed with 0.4 μg/ml of anti- MORC3 mouse mAb, followed by horseradish peroxidase (HRP) goat anti-mouse IgG (1: 7,500 dilution) (Southern Biotechnology, Birmingham, AL, USA) and developed using SuperSignal West Femto chemiluminescent substrate (Thermo Scientific, Barrington, IL, USA).

### Indirect immunofluorescence (IIF)

Immunofluorescent antinuclear/cytoplasmic antibodies (HEp-2 antinuclear antibody (ANA) slides) (INOVA Diagnostics, San Diego, CA, USA) were tested using a 1:80 dilution of human sera, or 4 μg/ml of anti-MORC3 mAb followed by DyLight 488 donkey IgG F(ab)'_2 _anti-human IgG or goat anti-mouse IgG (1:100 dilution) (Jackson ImmunoResearch, West Grove, PA, USA). Double staining was also performed using rabbit anti-PML antiserum (1:300 dilution) [18] and Alexa 568 goat anti-rabbit IgG (Invitrogen, Carlsbad, CA, USA).

### Statistical analyses

All parameters were analyzed by the Mann-Whitney test, or Fisher's exact test between groups using Prism version 5.0 d for Macintosh (GraphPad Software, Inc., La Jolla, CA, USA). Statistical significance was accepted at *P *< 0.05.

## Results

### Demographic data

Fifty-eight patients (43 females, 15 males) with a diagnosis of PM (25 cases), DM (27 cases) or overlap syndrome (6 cases) were studied. In the whole cohort, the patients' age was 52 ± 16 (mean ± standard deviation (SD)) years, the age at disease onset was 43 ± 17.4 years, and the mean follow-up was 56 months (range 1 to 288 months).

### Autoantibodies

**Figure 1:**
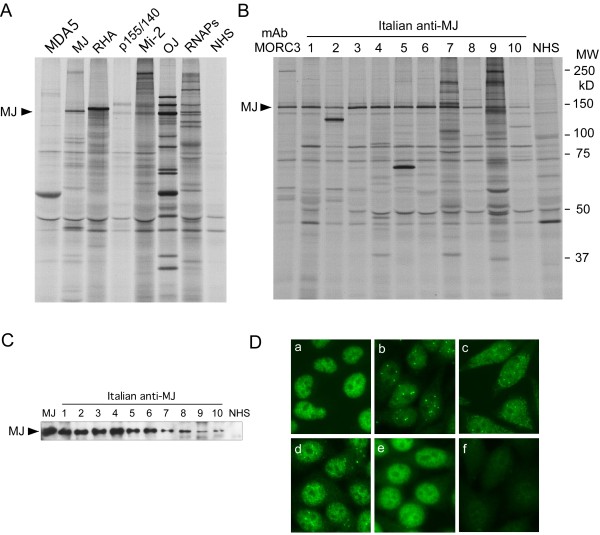
**Detection of anti-MJ antibodies**. **A. Immunoprecipitation of anti-MJ and other autoantibodies that recognize proteins of similar molecular weight**. ^35^S-methionine labeled K562 cell extract was immunoprecipitated by human sera and analyzed by 8% SDS-PAGE. Anti-MJ serum immunoprecipitates a ~140 kDa protein (arrow), which is different from the mobility of other known autoantigens. Reference sera for anti-MDA5, -MJ, -RNA helicase A (RHA), -p155/140, -Mi-2, OJ, and -RNA polymerases (RNAPs) are shown. NHS, normal human serum. **B. Immunoprecipitation of anti-MJ positive sera**. ^35^S-methionine labeled K562 cell extract was immunoprecipitated using mouse anti-MORC3 monoclonal antibody (lane mAb MORC3), human anti-MJ positive sera (lanes 1 to 10) or a normal human serum (NHS), and analyzed by 8% SDS-PAGE. MW, molecular weight marker. **C. IP-Western blot of MJ**. The identity of the 140 kDa protein as MJ/NXP-2/MORC3 was verified by IP-Western. The MJ protein is indicated with the arrow. MJ, anti-MJ reference serum, lanes 1 to 10, anti-MJ positive Italian samples. NHS, normal human serum. **D. Immunofluorescence staining of anti-MJ positive sera**. HEp-2 slides were stained with mouse anti-MORC3 monoclonal antibody **(a)**, human anti-MJ (+) sera **(b) **to **(e)**, or normal human serum **(f)**. Serum dilution, 1:80.

Prevalence of myositis-related autoantibodies by IP in total PM/DM, DM, and PM is summarized (Table 1). To our surprise, anti-MJ antibodies were the most prevalent specificity (10/58; 17%) in our PM/DM patients, followed by anti-Jo-1 (10%), -PM-Scl (10%), -U1RNP (7%), -p155/140 (5%), -SRP (5%), -EJ (3%), -Mi-2 (2%), and -OJ (2%). Anti-Ro and anti-Su were found in two cases (3%) each. In 22 patients (38%), no myositis-related autoantibodies were detected. In DM, no patient had anti-Jo-1 antibodies, while in PM it was the most common autoantibody specificity (0% vs 24%; *P *= 0.0087). Anti-MJ was found only in two cases in PM vs 30% in DM (*P *= 0.078). Thus, anti-MJ was common in DM, whereas anti-Jo-1 was found only in PM in our cohort. Candidates positive for anti-MJ were initially selected based on the IP of ~140 kD proteins compared with molecular weight markers and the mobility of other known autoantigens (Figure 1A). One of the known myositis autoantigen CADM140/MDA5, which migrates close to MJ, is not detectable in our IP system using K562 cells (Figure 1A, lane MDA5). IP of candidates were then run in SDS-PAGE along with anti-MORC3 mAb IP. If the mobility of a ~140 kD protein immunoprecipitated by the serum is considered same as that of the mAb to MORC3, sera were then tested by IP-Western to verify their identity as MJ. The 10 anti-MJ (+) were confirmed by 1) identical mobility of the 140 kDa protein with MORC3 recognized by mAb (Figure 1B); 2) IP- Western Blot (Figure 1C); and 3) positive results in antigen-capture ELISA (data not shown). None of anti-MJ (+) patients had other MSAs, consistent with previous observations [11], though the study in the Argentinian pediatric cohort reported 2/18 anti-MJ (+) cases with an additional autoantibody specificity [6]. NXP-2/MORC3 localizes to nucleus and nuclear dots, known as PML bodies. Number and intensity of PML bodies stained by mAb appear to vary depending on cell type, cell cycle and other factors [10]. In HEp-2 ANA slides, mAb to MORC3 stained nuclei in a fine speckled pattern with a few nuclear dots (Figure 1D, panel a). Six (three strong, three weak) of ten anti-MJ (+) sera showed PML bodies nuclear dots in indirect immunofluorescence, (Figure 1D, panels b to d), but PML bodies staining was not clear by other sera (Figure 1D, panel e), indicating that PML bodies immunofluorescence cannot be a good method for screening or confirmation of anti-MJ specificity.

**Table 1:** Autoantibody prevalence in a cohort of adult Italian patients with PM/DM.

	Total (*n *= 58)	DM (*n *= 27)	PM (*n *= 25)	Overlap syndrome (*n *= 6)
**Myositis-related antibodies**				

Anti-MJ	17% (10)	30% (8)^1^	8% (2)^1^	0

Anti-Jo-1	10% (6)	0^2^	24% (6)^2^	0

Anti-p155/140	5% (3)	7% (2)	0	17% (1) (DM-SLE-Sjögren's)

Anti-SRP	5% (3)	0	8% (2)	17% (1) (PM-RA)

Anti-EJ	3% (2)	4% (1)	4% (1)	0

Anti-Mi-2	2% (1)	5% (1)	0	0

Anti-OJ	2% (1)	0	4% (1)	0

Anti-PM/Scl	10%(6)	11% (3)	8% (2)	17% (1) (DM-SSc)

Anti-U1RNP	7% (4)	4% (1)	4% (1)	33% (2) (DM-SSc; PM-SLE)

Anti-Ro	3% (2)	0	8% (2)	0

Anti-Su	3% (2)	4% (1)	0	17% (1) (PM-SSc)

**Negative/unknown**	38% (22)	41% (11)	40% (10)	17% (1) (PM-SSc)

^1^*P *= 0.078; ^2^*P *= 0.0087 by Fisher's exact test. DM, dermatomyositis; PM, polymyositis; RA, rheumatoid arthritis; SLE, systemic lupus erythematosus. SSc, systemic sclerosis.

### Clinical and laboratory features

Since anti-MJ antibodies were the most common in PM/DM (17%) or in DM (30%), clinical features of 10 anti-MJ (+) vs 48 anti-MJ (-) cases were compared (Table 2). Anti-MJ (+) patients had younger age of disease onset (25.5 ± 13.8 vs 46.1 ± 15.9 years, mean ± SD, *P *= 0.0006 by Mann-Whitney) and age at initial visit (37.6 ± 12.0 vs 54.6 ± 14.8 years, *P *= 0.0023 by Mann-Whitney) compared with anti-MJ (-). Two anti-MJ (+) patients had pediatric onset DM. DM is more common in anti-MJ (+) vs (-) (80% vs 40%, *P *= 0.03) and no overlap syndrome patients were found in the anti-MJ group (0% vs 13%). In anti-MJ (+) patients, heliotrope rash (*P *= 0.01) and calcinosis (*P *= 0.09) were common, but none of them had heart involvement (0% vs 13%), interstitial lung disease (ILD) (0% vs 33%, *P *= 0.048), or malignancy (0% vs 8%). Myopathy in anti-MJ (+) patients responded well to therapy and elevated creatine phosphokinase (CPK) was not seen in any patient in the last visit (0% vs 25%). Thus, anti-MJ antibodies are associated with DM of young onset with heliotrope rash and calcinosis, without cardiac or lung involvement or overlapping features, and they respond well to therapy. Other clinical aspects, such as arthritis and Raynaud's phenomenon, were not significantly associated with anti-MJ antibodies (Table 2). Clinical characteristics of anti-MJ (+) DM compared to anti-MJ (-) DM were similar to those shown in Table 2, however, they did not reach statistical significance due to small numbers; heliotrope, 75% vs 47%; calcinosis 38% vs 21%; arthritis 25% vs 5%; heart involvement 0% vs 16%; and ILD 0% vs 32%.

**Table 2:** Demographic, clinical and laboratory features in anti-MJ (+) and -MJ (-) patients.

	Anti-MJ (+) *n *= 10	Anti-MJ (-) *n *= 48	*P* ^1^
**Demographic data**			

Male	40%	23%	

Mean age, ys (± SD)	37.6 (± 12)	54.6 (± 14.8)	0.0023

DM/PM/overlap (%)	80/20/0	40/48/13	DM 0.03

**Clinical and laboratory data**			

Heliotrope rash	60%	19%	0.01

Calcinosis	30%	8%	0.09

Facial erythema	60%	33%	ns

Gottron's papules	20%	19%	ns

Arthritis	20%	8%	ns

Raynaud's phenomenon	20%	27%	ns

Heart involvement	0%	13%	ns

Interstitial lung disease	0%	33%	0.048

Elevated CPK at last visit	0%	25%	ns

^1^*P *values are by Fisher's exact test except for mean age comparison by Mann-Whitney. CPK, creatine phosphokinase; DM/PM, dermatomyositis/polymyositis; ns, not significant; SD, standard deviation.

## Discussion

Anti-MJ antibodies were originally described as an abstract in 1997 in a subset of patients with JDM, characterized by severe muscle involvement [4]. Anti-MJ was reported in two juvenile DM studies [5,6], however, its detection in adult PM/DM was only in the form of an abstract [11] until recently, probably due to limited availability of the IP assay of this autoantibody [6]. A recent study in an adult Japanese cohort of patients with inflammatory myopathy reported 1.6% (8/507) prevalence of anti-MJ antibodies [12]. The most striking aspect observed in this cohort is that anti-MJ positive patients have higher prevalence of malignancy [12]. Lack of malignancy in anti-MJ positive patients in our cohort may be related to their young age (average 37.6, range 24 to 56 with only 2/10 over age 50) compared to anti-MJ (+) patients with malignancy reported (ages 54 to 68) [12]. This may be similar to a strong association of anti-p155/140 with malignancy in middle- to old-age DM but not in children or young adults [19,20].

Anti-MJ antibodies are the most prevalent specificity (30% in DM and 17% in PM/DM) in this Italian cohort, followed by other known MSAs, such as anti-Jo-1 and -Mi-2 (Table 1). The prevalence of anti-MJ in the present study is similar to two juvenile DM studies performed in Argentine [6] and UK/Ireland [5]. However, our study is the first report on high prevalence of anti-MJ antibodies in a cohort of adult patients with PM/DM vs only 1.6% to 3% in PM/DM or 1.6% to 6% in DM in other reports [11,12]. This could be due to different ethnic background, techniques, or other reasons. Genetic and/or environmental factors within Caucasians may be important variables, since the prevalence of anti-MJ in American Caucasian adults is low [21]. In fact, we have recently collected data on the different prevalence of autoantibodies in American Caucasian patients followed in our center, and only 3/73 (4%; all three DM cases) were anti-MJ (+) [21]. Two additional anti-MJ (+) cases were identified in a male Hispanic and a male African American DM patient. All of the five American anti-MJ (+) cases have DM features similar to those identified in the Italian cohort. Significant differences within Caucasians in prevalence of scleroderma-related autoantibodies are also reported [22]. In the same way, another unusual result from our study is the low prevalence of anti-Jo-1 antibodies in the Italian cohort (10%; 6 PM cases), and this is different from the American Caucasian cohort in which anti-Jo-1 was the most frequent specificity [21]. It is not clear why these antibodies have such a different prevalence according to the cohorts studied, but also in this case we can hypothesize technical issues or factors involved in the disease pathogenesis.

The majority (8/10) of anti-MJ (+) patients has DM, consistent with data in previous cohorts [5,6,12]. Two of our patients with anti-MJ can be classified as juvenile onset DM, but even in the cases of adult onset, anti-MJ (+) patients have younger onset of DM, compared with anti-MJ (-) patients (Table 2). We were also able to identify specific clinical features in our anti-MJ (+) patients, who are characterized by severe skin disease and extensive calcinosis, in the absence of internal organ involvement. Myositis is usually well responsive to treatment, and CPK levels tend to normalize quickly after starting therapy (data not shown). These features are similar to those reported in a cohort of British JDM patients [5], but different from the results of another study in Argentinian juvenile myositis [6], even if the patients of this cohort were defined as 'Argentine Caucasian' due to the Spanish or Italian descent. This study shows that anti-MJ (+) Argentinian patients mainly had muscle contractures, atrophy and significant compromise of the functional status, which are not seen in our Italian cohort of adult PM/DM. The clinical expression of PM/DM associated with anti-MJ (+) antibodies is very peculiar when considering juvenile or adult onset of disease.

The target recognized by anti-MJ antibodies was identified as a protein called NXP-2 (also known as MORC3) [7], involved in transcriptional regulation and activation of the tumor suppressor p53, which prevents cell proliferation by inducing cellular senescence [8,10]. Previous IIF studies showed that NXP-2/MORC3 localizes to both PML nuclear bodies and the nucleoplasm [10]. In the IIF study of our cohort of adult Italian PM/DM, only 60% of anti-MJ (+) had nuclear dots consistent with PML bodies. One possible explanation for this partial positivity is that the MJ antigens in PML bodies are not well recognized by certain human autoantibodies, maybe due to denaturation by fixation of ANA slides, poor reactivity with post-translationally modified forms that accumulate in PML bodies [8], interference by other proteins, low titer of the antibodies, or other reasons. The link between NXP-2/MORC3, autoantibody production and disease development is not clear yet. The report of NXP-2 as a SUMO (small ubiquitin-like modifier) target with a possible role in SUMO-mediated transcriptional repression [23] is an interesting link with disease mechanisms, because antibodies to SUMO-1 activating enzyme (SAE) have been found in DM [24], and the p155 and Mi-2 antigens could also be involved in transcriptional regulation [25]. Nevertheless, none of anti-MJ positive patients had anti-SAE antibodies that immunoprecipitate 90 kD and 40 kD proteins [24] (Figure 1B).

## Conclusions

In summary, anti-MJ antibodies are the most prevalent specificity in our cohort of adult Italian PM/DM patients. This autoantibody is associated with specific clinical features in DM patients, similar to those identified in British patients with JDM, such as severe calcinosis, skin disease but no internal organ involvement [5]. IIF cannot be considered as a screening or verification method to identify anti-MJ (+) samples, as only some anti-MJ (+) samples show a pattern consistent with PML nuclear bodies. Nevertheless, anti-MJ should be considered when PML body staining is seen in PM/DM patients. Further studies in various ethnicities and with larger cohorts of patients with anti-MJ antibodies are required to better understand clinical associations, etiopathogenesis and mechanisms of disease development.

## Abbreviations

ANA: antinuclear antibody; CPK: creatine phosphokinase; ELISA: enzyme-linked immunosorbent assay; HRP: horseradish peroxidase; IgG: immunoglobulin G; IIF: indirect immunofluorescence; ILD: interstitial lung disease; IP: immunoprecipitation; JDM: juvenile dermatomyositis; kD: kiloDaltons; mAb: monoclonal antibody; MSAs: myositis-specific autoantibodies; OD: optical density; PM/DM: polymyositis/dermatomyositis; PML: promyelocitic leukemia; SAE: small ubiquitin-like modifier-1 activating enzyme; SD: standard deviation; SDS-PAGE: sodium dodecyl sulfate-polyacrylamide gel electrophoresis; SUMO: small ubiquitin-like modifier.

## Competing interests

The authors declare that they have no competing interests.

## Authors' contributions

AC, SJR, BAP, JYFC, EKLC, and MS carried out the immunoassays. AC, IC, FF, EKLC, and MS designed the study. MS performed the statistical analysis. AC, MF, MT, IC, FF, MQ, and AT enrolled patients for the study, collected information and maintained the database. AC, EKLC, and MS drafted the manuscript. All authors read and approved the final manuscript.
